# H7N9 Influenza Virus Containing a Polybasic HA Cleavage Site Requires Minimal Host Adaptation to Obtain a Highly Pathogenic Disease Phenotype in Mice

**DOI:** 10.3390/v12010065

**Published:** 2020-01-05

**Authors:** Mable Chan, Anders Leung, Tamiko Hisanaga, Brad Pickering, Bryan D. Griffin, Robert Vendramelli, Nikesh Tailor, Gary Wong, Yuhai Bi, Shawn Babiuk, Yohannes Berhane, Darwyn Kobasa

**Affiliations:** 1 Special Pathogens Program, National Microbiology Laboratory, Public Health Agency of Canada, 1015 Arlington Street, Winnipeg, MB R3E 3R2, Canada; mable.hagan@canada.ca (M.C.); anders.leung@canada.ca (A.L.); bryan.griffin@canada.ca (B.D.G.); robert.vendramelli@canada.ca (R.V.); nikesh.tailor@canada.ca (N.T.); 2 National Centre for Foreign Animal Disease, Canadian Food Inspection Agency, Winnipeg, MB R3E 3M4, Canada; tamiko.hisanaga@canada.ca (T.H.); bradley.pickering@canada.ca (B.P.); shawn.babiuk@canada.ca (S.B.); yohannes.berhane@canada.ca (Y.B.); 3 Department of Medical Microbiology and Infectious Diseases, University of Manitoba, 745 Bannatyne Avenue, Winnipeg, MB R3E 0J9, Canada; 4 Institut Pasteur of Shanghai, Chinese Academy of Sciences, Life Science Research Building 320 Yueyang Road, Xuhui District, Shanghai 200031, China; garyckwong@hotmail.com; 5 Département de microbiologie-infectiologie et d’immunologie, Université Laval, 1050 avenue de la Médecine, QC G1V 0A6, Canada; 6 CAS Key Laboratory of Pathogenic Microbiology and Immunology, Institute of Microbiology, Center for Influenza Research and Early-warning (CASCIRE), Chinese Academy of Sciences, Beijing 100101, China; beeyh@im.ac.cn

**Keywords:** HPAI, H7N9, influenza virus, mammalian adaptation, mice, polybasic HA

## Abstract

Low pathogenic avian influenza (LPAI) H7N9 viruses have recently evolved to gain a polybasic cleavage site in the hemagglutinin (HA) protein, resulting in variants with increased lethality in poultry that meet the criteria for highly pathogenic avian influenza (HPAI) viruses. Both LPAI and HPAI variants can cause severe disease in humans (case fatality rate of ~40%). Here, we investigated the virulence of HPAI H7N9 viruses containing a polybasic HA cleavage site (H7N9-PBC) in mice. Inoculation of mice with H7N9-PBC did not result in observable disease; however, mice inoculated with a mouse-adapted version of this virus, generated by a single passage in mice, caused uniformly lethal disease. In addition to the PBC site, we identified three other mutations that are important for host-adaptation and virulence in mice: HA (A452T), PA (D347G), and PB2 (M483K). Using reverse genetics, we confirmed that the HA mutation was the most critical for increased virulence in mice. Our study identifies additional disease determinants in a mammalian model for HPAI H7N9 virus. Furthermore, the ease displayed by the virus to adapt to a new host highlights the potential for H7N9-PBC viruses to rapidly acquire mutations that may enhance their risk to humans or other animal species.

## 1. Introduction

The pandemic potential of avian influenza A (H7N9) viruses has risen substantially since its first emergence as a low pathogenic avian influenza (LPAI) virus in 2013. Risk assessment by the US Centers for Disease Control and Prevention characterizes these viruses to pose a moderate to high pandemic threat to humans [1,2]. As of June 2019, avian influenza A (H7N9) viruses had caused six epidemic waves of human disease in China, totalling 1568 laboratory confirmed cases and 616 deaths (~40% case fatality rate) [3]. For the first four epidemic waves, only LPAI H7N9 viruses circulated and caused asymptomatic or mild disease in poultry [4]. The lack of observable disease in poultry populations posed a considerable challenge in prevention and control of the disease, and highlighted the need for active surveillance for these viruses.

During the fifth epidemic wave in 2016–2017, several concerning changes were discovered about the LPAI H7N9 viruses. Approximately 21% of epidemic H7N9 virus samples obtained from humans and the environment contained a four amino acid insertion (K-R-T-A) in the hemagglutinin (HA) protease cleavage site, a mutation that is characteristic of high pathogenic avian influenza (HPAI) viruses [2,5,6]. These HPAI H7N9 viruses demonstrated greater morbidity and mortality in poultry [6]. Although this epidemic wave resulted in the greatest number of confirmed cases of human H7N9 infections (> 700 cases) with 28 of the cases caused by HPAI H7N9 infections [7,8], the overall disease severity was similar to previous patients infected with LPAI H7N9 [4,9,10]. However, the geographical spread of these viruses in China extended the furthest during this epidemic, with new areas reporting cases of H7N9 infections for the first time [4,6,11]. Since 2013, the HA genes of LPAI H7N9 viruses have evolved into two main lineages, the Yangtze River Delta and Pearl River Delta lineages [12], and studies have shown that H7N9 vaccines generated using A/Anhui/1/2013 (LPAI H7N9) induced antibodies that provided moderate recognition of recent HPAI H7N9 viruses [5].

To control the spread of H7N9 viruses, the Government of China launched a mandatory vaccination program for avian influenza in July of 2017. By November 2017, a newly developed bivalent H5/H7 inactivated vaccine was used to vaccinate over 282 million domestic poultry in China [13]. A series of strategies including the closure or management of live poultry markets (LPMs) [14] and vaccination resulted in significant reduction in the number of H7N9 cases or outbreaks detected at LPMs or poultry farms and a corresponding decrease in H7N9 infections in humans, specifically in the Guangdong Province [13]. It is important to note though that vaccination does not result in sterilizing immunity in vaccinated birds, therefore, continued replication and antigenic drift of these viruses in bird populations continues to pose a threat of a future re-emergence of H7N9 viruses.

The recent evolution of LPAI H7N9 to HPAI H7N9 viruses emphasizes the need to better understand the potential of these viruses to cause severe disease in humans, and what contributes to their pathogenicity. While the main difference between these two viruses is the presence of a polybasic cleavage site (PBC) in HA, whether this alone is required for increased pathogenicity in mice or if other factors are needed was the focus of our study. We investigated the pathogenesis of HPAI H7N9 viruses in the mouse model and identified the determinants of pathogenicity by reverse genetics. We show that introduction of a PBC HA into LPAI H7N9 virus (A/Anhui/1/2013, H7N9-PBC) alone did not elicit a high pathogenic phenotype in mice upon initial exposure to the virus. However, a single passage of the reverse genetics generated H7N9-PBC virus in mice was necessary and sufficient for it to become highly pathogenic and lethal, pointing to a need for host adaptation in the H7N9-PBC virus to achieve its high pathogenic potential in this host. Sequencing of the passaged virus revealed critical mutations in addition to the PBC cleavage site that contributed to high pathogenicity of these viruses.

## 2. Materials and Methods

### 2.1. Cells

Human embryonic kidney cells GripTite 293 MSR (293GT, Life Technologies, Carlsbad, CA, USA) and Madin Darby Canine Kidney (MDCK, ATCC, Manassas, VA, USA) cells were grown in either Dulbeco’s minimum essential medium (DMEM, HyClone, GE Healthcare Life Sciences, Logan, Utah, USA) or minimum essential medium (MEM, HyClone) supplemented with 5% fetal bovine serum (FBS, HyClone) and 1X L-glutamine (L-Glu, Gibco, Life Technologies, Grand Island, NY, USA), respectively. Human lung epithelial cells (A549, ATCC) were cultured in DMEM supplemented with 5% FBS and L-Glu. Mouse lung cells (KLN 205, ATCC) were cultured in MEM supplemented with 10% FBS and L-Glu. Chicken fibroblast cells (DF-1, ATCC) were cultured in DMEM supplemented with 10% FBS and L-Glu. All cells were grown in a humidified incubator with 5% CO_2,_ at either 37 °C (293GT, MDCK, A549, and KLN 205 cells) or 39 °C (DF-1 cells).

### 2.2. Construction of Recombinant H7N9 Viruses

The sequence of the clinical isolate of A/Anhui/1/2013 (H7N9) was obtained using sequences uploaded onto the Global Initiative on Sharing All Influenza Data (GISAID) database (EPI_ISL_138739). A recombinant virus was fully synthetically generated using overlapping primers to construct all gene sequences. The gene2oligo website (http://berry.engin.umich.edu/gene2oligo/) was used to design primers for the generation of full-length influenza genes using a hybridization size of 40 base pairs. The eight full length influenza virus genes (HA, NA, M, NS, NP, PB1, PB2, PA) were amplified using iProof DNA polymerase (Bio Rad, Hercules, CA, USA). These genes were then cloned into the pPol vector that contains the RNA polymerase I driven reverse genetics system as described previously [15] by in-fusion cloning (Takara Bio, Mountain View, CA, USA).

The HA gene was mutated using PrimeSTAR Max (Takara Bio) DNA polymerase to introduce a PK-**R-R-R-R↓** PBC site by PCR [16], and the mutated PCR amplicon was inserted back into the pPol vector containing HA by in-fusion cloning. Helper plasmids encoding the viral nucleoprotein (NP) and polymerase genes (PB1, PB2 and PA) were cloned into the pCAGGS eukaryotic expression system. Amino acid mutations in HA (A452T, H7 HA numbering with the signal peptide included), PA (D347G), and PB2 (M483K) were also inserted into each respective pPol vector by PCR using PrimeSTAR Max and in-fusion cloning.

### 2.3. Virus Rescue

To rescue the recombinant viruses 1 µg of each helper plasmid along with 100 ng of each RNA pol I plasmid for each gene were transfected with 10 µL LT1 transfection reagent (Mirus Bio, Madison WI, USA) in 200 µL OPTI-mem (HyClone) in 293GT cells. After 48 h, the supernatant was collected and treated with 1 µg/mL of N-tosyl-L-phenylalanine chloromethyl ketone (TPCK) treated trypsin (Sigma Aldrich, ON, Canada) for 30 min at 37 °C, blind passaged onto MDCK cells, and harvested after the appearance of cytopathic effect (CPE) 48–72 h later. Two versions of A/Anhui/1/2013 (H7N9) were rescued by reverse genetics, the wild-type version (H7N9-RG) and a version containing the PBC HA (H7N9-PBC). To generate viral stocks, MDCK cells in T150 cm^2^ flasks were washed with phosphate buffered saline (PBS), and infected with a low multiplicity of infection (MOI) of virus diluted in MEM supplemented with 0.1% bovine serum albumin (BSA, Gibco), L-Glu, 1 µg/mL TPCK treated trypsin (Sigma Aldrich, ON, Canada) for 48–72 h. Viruses were harvested when 80%–90% CPE was observed, and stocks were titered by plaque assays.

### 2.4. Virus Titration, Growth Kinetics and Trypsin Assays

Viral titers in samples were measured by median tissue culture infectious dose (TCID50) or plaque assays using MDCK cells. Samples were serially diluted 1:10 in MEM supplemented with 0.1% BSA, L-Glu, and 1 µg/mL TPCK trypsin, and 100 µl of each virus dilution was added in triplicate to MDCK cells in 96-well plates that were washed 1X with PBS. Plates were incubated at 37 °C with 5% CO_2_ and the presence of CPE was determined after 48–72 h. TCID50/mL was determined using the Reed and Muench method [17]. For plaque assays, 200 µl of each sample dilution was added to MDCK cells in 12-well plates that were washed 1X with PBS. Virus was adsorbed for 1 h at 37 °C and then removed, cells were overlayed with 1% SeaPlaque agarose (Lonza, Rockland, ME, USA) in 1X MEM supplemented with 0.1% BSA, L-Glu and 1 µg/mL TPCK trypsin. Plaques were counted after 48–72 h, and titer was reported as plaque forming units (PFU) per mL.

To determine viral growth in MDCK, A549, KLN 205 and DF-1 cells, each cell line was infected in triplicate with a MOI of 0.001 in MEM supplemented with 0.1% BSA, L-Glu, and 0.5 µg/mL TPCK trypsin. Assays were performed at 37 °C on MDCK, A549 and KLN 205 cells, and 39 °C in DF-1 cells. The concentration of trypsin was reduced due to trypsin sensitivity exhibited by DF-1 cells. Supernatant was collected at 24 and 48h post-infection (pi) for titration by TCID50 assay as described.

For growth without trypsin assays, viruses were grown on MDCK cells with or without the presence of 1 µg/mL TPCK trypsin in culture medium. Samples that were grown without trypsin were first trypsin activated before titration by the addition of 1 µg/mL TPCK trypsin to each sample, followed by incubation at 37 °C for 30 min. Samples that were grown in the presence of trypsin were also incubated at 37 °C for 30 min. After activation, samples were titrated by TCID50 assay.

### 2.5. Ethics Statement

This animal work was performed as described in protocol H-16-012 (396 mice total) approved on 19 October 2016, by the Animal Care Committee at the Canadian Science Center for Human and Animal Health. This protocol was carried out in accordance with the guidelines set by the Canadian Council on Animal Care.

### 2.6. Mouse Experiments

Four to five week old female BALB/c mice were ordered from Charles River (Montreal, QC, Canada) and infected following a week acclimatization period in our biosafety level-4 (BSL-4) facility. For all infections, mice were anesthetized using isoflurane and infected intranasally (IN) with 50 µL of virus diluted in MEM supplemented with 0.1% BSA. To determine the mouse median lethal dose (MLD_50_) of H7N9 influenza viruses, 10-fold serial dilutions of each virus was performed and groups of mice (*n* = 5 to 6) were infected IN with each dilution and monitored daily for weight loss and survival. Mice were euthanized when ≥25% weight loss was reached as outlined in our approved Animal Use Document. The MLD_50_ value was calculated using the method of Miller and Tainter [18].

For mouse passaging, mice (*n* = 3) were infected with 7 × 10^5^ PFU of either H7N9-RG or H7N9-PBC. Animals from each group were euthanized on day 4 pi, lungs were removed and homogenized using the TissueLyser II (QIAGEN, Hilden, Germany) for 6 min at 30 Hz with 500 µL of MEM medium, and cell debris was pelleted by centrifugation at 1500 x g for 10 min. These lysates were considered mouse passaged 1 virus (mP1) for each group (H7N9-RG mP1 or H7N9-PBC mP1). Next, 50 µL of each homogenized lung tissue was used to infect naïve BALB/c mice (*n* = 2 for each lung lysate, total *n* = 6/virus), respectively via the IN route. Mice were monitored for 6 days pi, euthanized and lungs were collected and homogenized, producing mouse passaged 2 viruses (H7N9-RG mP2 and H7N9-PBC mP2). Mice (*n* = 3) were infected IN with the mP2 viruses and monitored for weight loss and survival. Viruses at each passage were titered by TCID50 or by plaque assay.

To examine the tissue distribution of H7N9-RG mP2 and H7N9-PBC mP2 viruses in mice, a serial sacrifice study was performed. Mice (*n* = 5/sacrifice day) were infected IN with 1 × 10^2^ PFU of either H7N9-RG mP2 or H7N9-PBC mP2 virus and sacrificed on days 3, 6 and 8 pi to collect blood, heart, spleen, kidney, intestine and lung tissues. Tissues were weighed, homogenized and viral loads were measured by RT-qPCR using primers and probes detecting the influenza viral M gene [19]. Viral RNA was extracted using the QIAamp Viral RNA Mini kit (QIAGEN), and levels of influenza virus M were quantified using Light Cycler 480 RNA Master Hydrolysis kit (Roche, Mannheim, Germany). Genome equivalents were calculated based on a standard curve generated using the same pPol vector containing the influenza virus M gene used for virus rescue. Samples that were positive by RT-qPCR were also titrated by TCID_50_ assay.

### 2.7. Next-Generation Sequencing (NGS)

Full-length influenza A virus gene segments were amplified directly from total RNA that was extracted using the RNeasy Mini Plus Kit or Viral RNA Mini Kit (QIAGEN) using previously described universal influenza primers [19]. Purified gene segments were subsequently quantified using a Biodrop Touch UV spectrophotometer (SERVA, Biophoretics, Sparks, NV, USA). Sequencing library construction was performed using the Ion Xpress™ Plus Fragment Library Kit (Life Technologies) utilizing a 5 min shearing time. IonXpress™ Barcode adapters (Life Technologies) were applied to each full genome isolate or individual gene segments when required. Size selection of each barcoded library was performed on a PIPPIN-Prep using 2% agarose gels (Sage Science, Toronto, ON, Canada). Size-selected libraries were qualitatively assessed using the Agilent High Sensitivity DNA Kit and Agilent 2100 Bioanalyzer (Agilent Technologies Canada Inc., Mississauga, ON, Canada). RT-qPCR was performed with a 7500 Fast Real-Time PCR System (Applied Biosystems) and the Ion Library Quantitation Kit (Life Technologies) to determine template dilution factor for emulsion PCR. Barcoded libraries were pooled and a DNA template was prepared for sequencing using the Ion PGM™ Template OT2 Reactions 200 kit (Life Technologies) with the Ion OneTouch™2 System with ES for ion sphere particles (ISP) enrichment. Quality control of ion sphere particles (ISP) was performed using Ion Sphere™ Quality Control Kit and a Qubit fluorometer (Life Technologies). Sequencing was performed with an Ion Torrent PGM™ instrument using an Ion 314™ Chip Kit v2 (Life Technologies) and an Ion PGM ™Hi-Q™ Sequencing Kit. Data analysis was performed using DNAstar SeqMan NGen^®^ software (Madison, WI, USA), DNAstar Core suite and BLAST server at National Center for Biotechnology Information (NCBI). During library build, Agencourt AMPure XP Reagent (Beckman Coulter, Mississauga, ON, Canada) was used for purification when required following all manufacturer protocols.

### 2.8. Desialylation and Resialylation of Turkey Red Blood Cells

A 10% suspension of turkey red blood cells (TRBCs) was incubated at a ratio of 1:2 with receptor destroying ezyme (RDE II, Denka Seiken, Hardy Diagnostics, Santa Maria, CA, USA) for 1 h at 37 °C. Cells were pelleted by centrifugation at 2000 x rpm for 5 min, and washed 3X with 50 mL of PBS. For every 100 µL of 10% TRBCs, 4 mU of α-2,3-sialyltransferase from Pasteurella multocida (Sigma-Aldrich, Oakville, ON, Canada) or 4 mU α-2,6-sialyltransferase from Photobacterium damsela (Sigma-Aldrich) and 1.5 mM cytidine-5-monophospho-N-acetylneuraminic acid sodium salt (CMP, Sigma-Aldrich) was added, or mock-treated with an equivalent volume of PBS. TRBCs were incubated at 37 °C for 4 h with gentle mixing every 15 min. Cells were washed 3X with PBS and resuspended as a 1.5% suspension and stored at 4 °C. On the next day, 2-fold serial dilutions of virus was prepared in PBS. Using V-bottom plates (Corning, NY, USA), 50 µL of each virus dilution was added to each well followed by addition of 50 µL of a 1.5% suspension of TRBCs (supplemented with 3% BSA) that were either untreated (native), PBS mock-treated, or resialylated with either α-2,3- or α-2,6-linked sialic acids (SA). Hemagluttination (HA) titers were read after 30 min at room temperature and reported as the reciprical of the lowest dilution showing complete agglutination. This procedure is a modification of the methods described in [20,21].

### 2.9. Statistics

MLD_50_ with 95% confidence interval (CI) values was calculated using the method of Miller and Tainter [18]. Comparison of viral titers was determined by two-way ANOVA with Bonferroni post-tests using GraphPad Prism 5 software (San Diego, CA, USA), results were considered statistically significant when *p*-value < 0.05.

## 3. Results

### 3.1. H7N9 Viruses Containing a Polybasic HA Cleavage Site Requires a Single Passage in Mice to Exhibit High Pathogenicity

**Figure 1:**
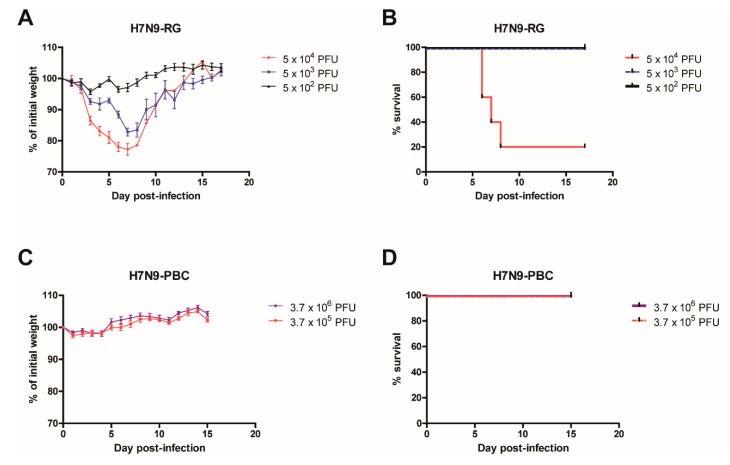
Virulence comparison in mice infected with reverse genetics generated influenza A/Anhui/1/2013 (H7N9-RG) virus or a version containing a polybasic cleavage site in HA (H7N9-PBC). Groups of mice (*n* = 5) were infected intranasally with 10-fold serial dilutions of H7N9-RG virus, starting at a dose of 5 × 10^4^ PFU. Weight loss (**A**) and survival (**B**) of H7N9-RG infected mice were measured for 17 days post-infection. For H7N9-PBC virus, groups of mice (*n* = 6) were infected intranasally with either 3.7 × 10^6^ or 3.7 × 10^5^ PFU of virus, and weight loss (**C**) and survival (**D**) of the mice are shown.

To investigate the contribution of a PBC site towards the pathogenicity of H7N9 influenza viruses, we used a PBC site that naturally emerged in a large outbreak of H7N7 virus in the Netherlands in 2003 [16]. The sequence of this PBC site, PEIPKRRRR↓, was inserted into A/Anhui1/2013 (H7N9-RG) background virus to generate a H7N9-PBC virus. Initial introduction of the reverse genetics engineered H7N9-RG virus and H7N9-PBC virus into BALB/c mice revealed dramatic differences in the disease severity of the animals. The MLD_50_ for each virus was determined by infecting groups of mice (*n* = 5 to 6) intranasally (IN) with doses ranging from 5 x 10^3^ to 5 x 10^5^ PFU of H7N9-RG virus, or 3.7 × 10^5^ to 3.7 × 10^6^ PFU of H7N9-PBC virus. Mice infected with the highest dose of H7N9-RG virus demonstrated rapid weight loss and 80% of the mice succumbed to infection by day 7 pi (Figure 1A,B). From these results, H7N9-RG has a MLD_50_ of 2.04 × 10^4^ PFU. In contrast, mice infected with the highest dose of H7N9-PBC virus, a dose that is 7.4-fold higher than H7N9-RG, showed no apparent weight loss or clinical signs of disease (Figure 1C,D). Based on the titer of H7N9-PBC stock we generated, this was the highest dose that we were able to test. As a result, the MLD_50_ for H7N9-PBC virus is greater than 3.7 × 10^6^ PFU.

**Figure 2:**
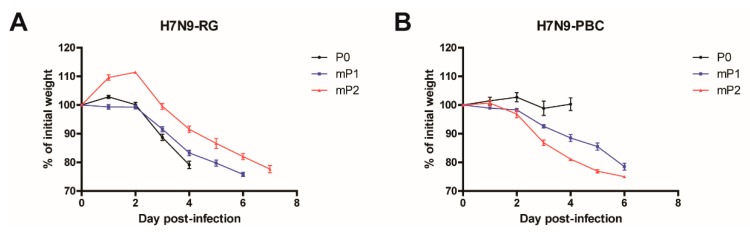
Pathogenesis of influenza A (H7N9) viruses containing a polybasic HA cleavage site upon serial passaging in mice. Mice (*n* = 3) were infected with 7 x 10^5^ PFU of either (**A**) A/Anhui/1/2013 (H7N9-RG, P0) or (**B**) A/Anhui/1/2013 (H7N9) containing a polybasic HA cleavage site (H7N9-PBC, P0). Mice were sacrificed on day 4 post-infection and lung homogenates from each mouse was collected for passaging. For the first passage of H7N9-RG and H7N9-PBC viruses in mice (mP1), groups of mice (*n* = 6) were infected with 50 µL of lung homogenates collected from P0 infected mice, respectively. For the second passage (mP2), three mice from each mP1-infected group were sacrificed on day 6 post-infection, and lung homogenates were collected and used to infect mice (*n* = 3). Mean weight loss (%) ± standard error of mean of infected mice are shown.

To determine whether viral adaptation of the H7N9-PBC virus is required to elicit a pathogenic phenotype in mice, we investigated the effect of serial passaging the virus in mice on pathogenicity. Groups of mice (*n* = 3) were infected IN with 7.5 × 10^5^ PFU of each virus (H7N9-RG or H7N9-PBC). Similar to the results in Figure 1, initial infection of mice (P0) with H7N9-RG virus resulted in rapid weight loss in comparison to H7N9-PBC infected mice that showed minimal weight loss (Figure 2A,B). On day 4 pi, these mice were sacrificed and lung homogenates were collected for subsequent passaging. For the first mouse passage (mP1), lung homogenate from each of the three mice infected with H7N9-RG or H7N9-PBC was used to infect a new set of naïve mice (*n* = 6). After one passage, H7N9-RG mP1 infected mice displayed similar weight loss and disease progression as H7N9-RG P0 infected mice. Surprisingly, mice infected with H7N9-PBC mP1 viruses resulted in dramatic weight loss accompanied by the appearance of clinical signs of disease, similar to that observed for the H7N9-RG-P0 and –mP1-infected mice.

To determine the viral dose given to each mouse during virus passaging, back titration of the virus contained in each lung homogenate was conducted by TCID_50_ assay. For the first passage, mice were infected with 1.4 × 10^4^ TCID_50_ of H7N9-RG mP1 or 7.9 × 10^3^ TCID_50_ of H7N9-PBC mP1. On average, mice received an approximately 1.8-fold higher dose of H7N9-RG mP1 compared to H7N9-PBC mP1 during the first passage. Although a lower dose of H7N9-PBC mP1 was administered to the mice, this single passage of H7N9-PBC was sufficient to abruptly change it from an initially non-pathogenic virus to one with increased virulence sufficient to cause severe weight loss.

A second passage of H7N9-RG and H7N9-PBC viruses was conducted to confirm the change in phenotype observed for at least the H7N9-PBC group, and to verify whether the pathogenicity of these viruses remained the same or if it continues to increase. Lung homogenates from mice infected with H7N9-RG mP1 and H7N9-PBC mP1 were collected on day 6 pi and passaged for a second time in naïve mice (*n* = 3, mP2). Severe weight loss was similarly observed in mice infected with either H7N9-RG mP2 or H7N9-PBC mP2 viruses (Figure 2A,B). For H7N9-PBC mP2 infected mice, two of the three mice succumbed to disease by day 6 pi, and the last mouse required euthanasia due to ≥25% weight loss. This is in contrast to mice infected with H7N9-PBC mP1, where all six of the mice survived to day 6 pi, suggesting that the additional passage of the virus selected for a more pathogenic variant of H7N9-PBC. Interestingly, mice infected with H7N9-RG mP2 were all euthanized on day 8 pi, when they reached >25% weight loss. For the second passage, an average dose of 1.9 × 10^4^ TCID_50_ of H7N9-RG mP2 or 9.7 × 10^4^ TCID_50_ of H7N9-PBC mP2 viruses was given to the mice, corresponding to approximately 5-fold more H7N9-PBC mP2 virus compared to H7N9-RG mP2. Overall, a single passage of H7N9-PBC was necessary to elicit a highly pathogenic phenotype in mice.

### 3.2. Mouse-Passaged H7N9-PBC Virus Is More Virulent Compared to H7N9-RG Virus

**Figure 3:**
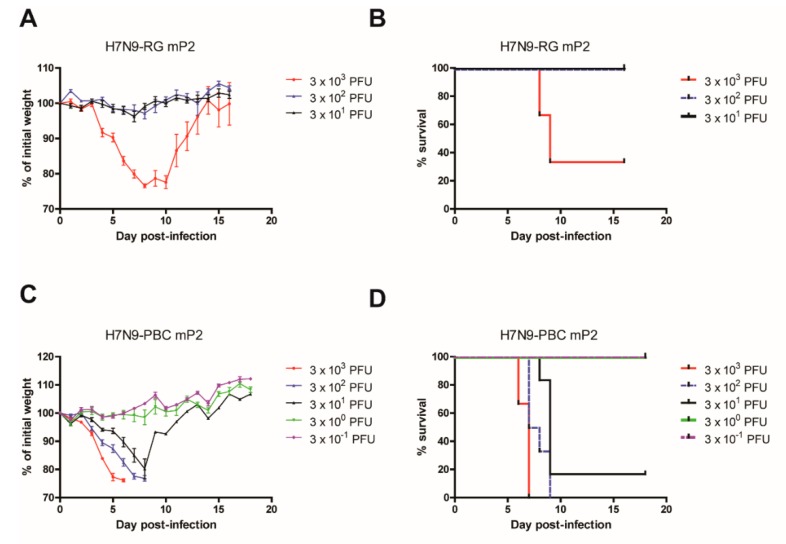
Determination of mouse median lethal dose (MLD_50_) for mouse-passaged H7N9 viruses. Groups of mice (*n* = 6) were infected intranasally with a dose range of 3 × 10^−1^ to 3 × 10^3^ PFU of either H7N9-RG mP2 (**A**,**B**) or H7N9-PBC mP2 (**C**,**D**) viruses. Mean percent weight loss ± standard error of mean (**A**,**C**) and survival curves (**B**,**D**) for each virus are shown.

To compare the virulence of mouse-passaged H7N9-RG to H7N9-PBC viruses the MLD_50_ value was determined for each mP2 virus. Mice (*n* = 6) were infected IN with a dose range of 3 × 10^−1^ to 3 x 10^3^ PFU per mouse. Mice infected with H7N9-RG mP2 showed weight loss in a dose dependent manner (Figure 3A) that correlated with survival rates of 100% at the lower doses of 3 × 10^1^ and 3 × 10^2^ PFU, and 33% at 3 × 10^3^ PFU (Figure 3B). From these results, the MLD_50_ of H7N9-RG mP2 was determined to be 1.368 × 10^3^ PFU (95% CI: 1.365–1.371 × 10^3^). For H7N9-PBC mP2 virus, the MLD_50_ was determined to be 7 PFU (95% CI: 6–8) (Figure 3C,D). This value is almost 200-fold lower than the MLD_50_ for H7N9-RG mP2 virus, emphasizing the importance of the PBC in HA as well as adaptation in the host, in conferring greater virulence to this mouse-adapted virus.

Virus passaging through mice will likely create mouse-adapted versions of the same virus with increased virulence in that animal model. Therefore, it was not surprising that mouse passaging of even the H7N9-RG viruses resulted in a more pathogenic version of this virus after two passages. The MLD_50_ value for H7N9-RG mP2 viruses was determined to be 1.37 × 10^3^ PFU compared to 2.04 × 10^4^ PFU for the non-passaged H7N9-RG virus; this is a decrease in MLD_50_ of approximately 15-fold after two passages. Although we observed an increase in pathogenicity of H7N9-RG virus with mouse passaging, it did not increase to the same extent that H7N9-PBC mP2 did, where the MLD_50_ of the latter decreased significantly by over 100,000-fold. H7N9-RG-mP2 viruses were also sequenced and mutations were found in NA (G389D, G389C), PB2 (M64R, D256G), and PB1 (A157V). The contribution of these changes to altered virulence in the mouse model for the H7N9-RG virus was not examined, but might be of interest in looking at changes that could contribute to mammalian host adaptation of H7N9 viruses.

### 3.3. Determinants of Pathogenicity of Mouse Passaged H7N9-PBC

To determine whether the H7N9-PBC virus acquired other genomic mutations during passaging in mice, the mouse-passaged virus was sequenced by next-generation sequencing. From these results, the percentage of variants within each viral sample was determined and within the H7N9-PBC mP2 virus we found three dominant amino acid mutations in the following genes: HA (A452T, 66%), PA (D347G, 68%) and PB2 (M483K, 65%) (Table 1). To confirm the importance of the three mutations identified in the mouse-passaged H7N9-PBC mP2 virus, this virus was recreated by reverse genetics (H7N9-PBC mP2-PB2/PA/HA) and used to infect mice. Furthermore, H7N9-PBC viruses containing individual or paired combinations of the three mutations identified in PB2, PA, and HA, were also produced to determine the contribution of each mutation to virulence in mice.

To compare the pathogenicity of the reverse genetics engineered viruses to the original mouse-passaged virus isolated from lung tissues, the MLD_50_ was determined for each virus (Table 1). Groups of mice (*n* = 6) were infected with a dose range of each virus, with the highest dose beginning at 1 × 10^4^ PFU followed by 10-fold serial dilutions until a MLD_50_ could be determined. In comparison to the original mouse-passaged H7N9-PBC mP2 virus obtained from lung tissues, the reverse genetics created version of this same virus behaved similarly in mice, having a comparable MLD_50_ value of 10 PFU. This confirms the importance of these three mutations in addition to the PBC in HA for the increased virulence observed in mice.

Of the three viruses with single mutations, the H7N9-PBC HA virus had the lowest MLD_50_ at 2.78 × 10^2^ PFU (95% CI: 2.65–2.91 × 10^2^) compared to an MLD_50_ of > 1 × 10^4^ PFU for the viruses containing either the single PB2 or PA mutations (Table 1). From the viruses with combinations of two mutations, H7N9-PBC viruses containing the HA mutation along with either PA (H7N9-PBC PA/HA) or PB2 (H7N9-PBC PB2/HA) had MLD_50_ values of 1.02 × 10^2^ PFU (95% CI: 1.01 × 10^2^ to 1.03 × 10^2^) and 2.22 × 10^2^ PFU (95% CI: 2.20 × 10^2^ to 2.23 × 10^2^), respectively. In contrast, the H7N9-PBC PB2/PA double mutant was significantly less pathogenic, having an MLD_50_ value > 1 × 10^4^ PFU. Although introduction of D347G (PA) or M483K (PB2) mutations alone appear to play no role in the pathogenicity of the H7N9-PBC virus at the doses tested, when combined with the A452T (HA) mutation a significant increase in pathogenicity was observed. However, the MLD_50_ values for the double mutants (mP2-PB2/HA or PA/HA) were still higher compared to the MLD_50_ of the reverse genetics H7N9-PBC mP2-PB2/PA/HA virus with the complete set of mutations. These results suggest that while the HA mutation is the most important contributor to virulence, the other mutations in PA and PB2 are necessary to achieve full virulence.

### 3.4. Detection of H7N9-PBC Viruses in Lungs and Other Tissues

A general attribute associated with the presence of a PBC site in HA is the ability to be cleaved by a set of alternative cellular proteases that have a widespread distribution in tissues, compared to monobasic HA cleavage sites recognized by trypsin-like proteases that reside primarily in the respiratory tract. To determine whether mouse-passaged H7N9-PBC virus (H7N9-PBC mP2) is able to spread and replicate in tissues other than the lungs a serial sacrifice study was performed. Groups of mice (*n* = 5) were infected IN with 1 × 10^2^ PFU of either H7N9-RG mP2 or H7N9-PBC mP2 virus, and tissues including the eyes, lungs, intestine, kidneys, spleen and heart were collected on days 3, 6, and 8 pi. Viral RNA was extracted from the homogenized tissues, and genome copies per gram of tissue or per ml of blood were determined by RT-qPCR detecting the M gene. For samples that were positive by RT-qPCR, live virus titers were determined by TCID_50_ assay.

From all the tissues tested, the lungs were the only tissues that were positive by RT-qPCR for both infected groups on all sample days, where the limit of detection for RT-qPCR was determined to be 50 genome copies/mL or /g (Table 2). Although all lung samples in the H7N9-RG mP2 infected group were positive by RT-qPCR, the mean Ct values for these samples were higher compared to the H7N9-PBC mP2 infected group on each respective sample day, pointing towards increased levels of replication in the H7N9-PBC mP2 infected mice. Measurement of infectious viral loads in the lungs by TCID_50_ assays confirmed these results, revealing consistently higher lung viral titers in H7N9-PBC mP2 infected animals across all sample days. Viral lung titers in the H7N9-PBC mP2 infected group were significantly higher on day 3 pi, having a difference in titer of 4.0-log_10_ (95% CI of diff. 1.3 to 6.7-log_10_, *p*-value < 0.01), in comparison to the H7N9-RG mP2 group (Table 2). Although the viral titers were not considered statistically significant on day 6 pi, with a difference of 1.1-log_10_ (95% CI of diff. −1.6 to 3.8-log_10_, *p*-value > 0.05), on day 8 pi the titers were significantly higher in the H7N9-PBC mP2 group compared to the H7N9-RG mP2 group (4.4-log_10_ difference, 95% CI of diff. 1.7 to 7.1-log_10_, *p*-value < 0.001). Overall, H7N9-PBC mP2 viruses replicated to higher titers in the lungs of infected mice compared to H7N9-RG mP2 virus.

Surprisingly, three mice in the H7N9-RG mP2 infected group were positive for viral RNA in the heart tissue on days 3 (*n* = 2) and 6 pi (*n* = 1) but remained negative for infectious virus when tested by TCID_50_ assay. Although many other tissues and blood from H7N9-PBC mP2 infected mice were positive for viral RNA on all sample days tested, like the H7N9-RG mP2 samples, no infectious virus was recovered by TCID_50_ assay. While it may appear that H7N9-PBC-mP2 viral RNA is detectable in a greater number of tissues and in the blood, and this suggests that viral replication may disseminate to tissues other than the lung, the lack of infectious virus recovered from these tissues could be due to the limit of detection of the assay and suggests that these tissues are not significant targets of viral infection.

### 3.5. Growth Kinetics of H7N9 Viruses in Mammalian Cell Culture

The significantly higher viral titers of H7N9-PBC mP2 found in the lungs of infected mice compared to H7N9-RG mP2 suggests that H7N9-PBC mP2 viral replication may exhibit increased kinetics in mammalian cells due to adaptation. Furthermore, differences in MLD_50_ values among the various single and double mutants of H7N9-PBC that were generated (Table 1) may also be related to replication efficiency, especially since two of the three mutations were found in the polymerase genes PB2 and PA. Comparison of the replication efficiency of these viruses was done by measuring virus replication of each virus in various mammalian cell lines including A549 (human lung), KLN 205 (mouse lung), DF-1 (chicken fibroblast), and MDCK (canine kidney) cells. Cells were infected with a low MOI of 0.001, to allow for multiple rounds of replication to occur, and viral titers were measured at 24 and 48 h pi by TCID_50_ assay. To statistically determine whether the viruses acquired the ability to replicate better in mouse cells with passaging, a two-way ANOVA analysis comparing all viral titers to replication in KLN 205 cells at each time point was performed.

**Figure 4:**
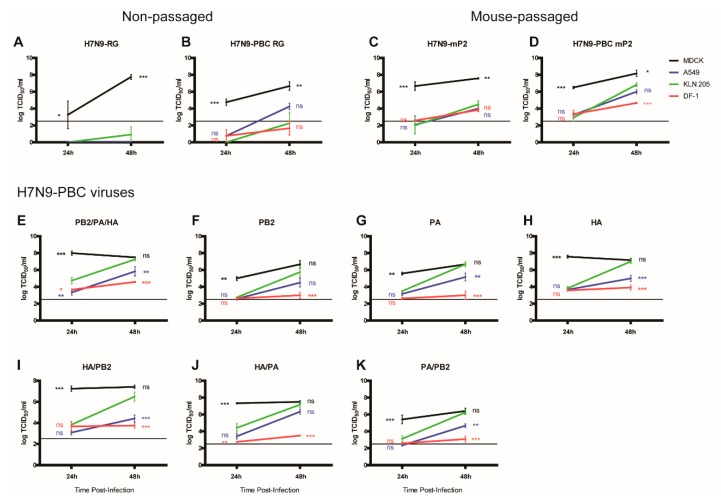
Growth of H7N9-PBC influenza viruses in mammalian cell lines. Viral replication was determined in Madin Darby Canine Kidney (MDCK), human lung (A549), mouse lung (KLN 205), and chicken fibroblast (DF-1) cell lines at 24 and 48 h post-infection with 0.5 µg/mL TPCK-trypsin supplemented into the culture medium. Viral titers were determined by TCID_50_ assay using MDCK cells with 1.0 µg/mL TPCK-trypsin in the medium. Non-passaged (**A**) H7N9-RG and (**B**) H7N9-PBC RG viruses were compared to their respective mouse-passaged (mP2) versions, (**C**) H7N9-mP2 and (**D**) H7N9-PBC mP2. Replication was also determined for the reverse genetics engineered H7N9-PBC mP2 virus (**E**, PB2/PA/HA), the recombinant H7N9-PBC viruses containing single mutations in (**F**) PB2, (**G**) PA, or (**H**) HA, or double mutations in (**I**) HA/PB2, (**J**) HA/PA, or (**K**) PA/PB2. Dotted line on each graph represents the limit of detection of the TCID_50_ assay. Mean viral titers and standard error of mean are shown, two-way ANOVA with Bonferroni post-tests was performed comparing all results to KLN 205 at each time point, ns = not significant, * or ** or *** denotes *p*-values of <0.05, or <0.01, <0.001, respectively.

In general, replication of H7N9-RG and H7N9-PBC RG was limited to MDCK cells at 24 h, with mean titers increasing to 8-log_10_ or 6.7-log_10_ TCID50/mL by 48h pi, respectively (Figure 4A,B). The presence of a PBC site in H7N9-PBC RG did not permit efficient replication in mouse or chicken cell types, however a mean titer of 4.3-log_10_ TCID50/mL was detected in human cells at 48h pi. Interestingly, mouse adaptation of these two viruses resulted in better replication overall in all cell lines and at both time points tested (Figure 4C,D). Although levels of MDCK replication was similar between the two mouse-adapted viruses, replication in human, mouse and chicken cells could be detected above the limit of detection at 24h pi for H7N9-PBC mP2 virus only. Furthermore, H7N9-PBC mP2 titers in mouse cells (6.8-log_10_ TCID_50_/_mL_) and human cells (6.0-log_10_ TCID_50_/_mL_) at 48h pi were higher compared to ~4.0-log_10_ TCID_50_/_mL_ reached in both cell types for H7N9-mP2. These results suggest that the PBC site along with the accompanying mutations in PB2, PA and HA are contributing to increased viral replication by H7N9-PBC mP2.

To determine which mutations in PB2, PA, or HA in H7N9-PBC mP2 virus play a role in the ability to replicate in different mammalian cell lines, viral replication of the reverse genetics generated H7N9-PBC RG mP2 virus (Figure 4E) and all of the H7N9-PBC viruses with single and double adaptation mutations were also compared (Figure 4F–K). For this set of viruses, the greatest replication was observed in MDCK cells followed by mouse and then human cells. Chicken cells supported the lowest levels of viral replication. In general, all of the mutant H7N9-PBC viruses containing either single or double mutations (Figure 4F–K), replicated to higher titers at 24h pi compared to the non-passaged version H7N9-PBC RG (Figure 4B). In addition, viruses containing the HA mutation (Figure 4E,H,I,J) replicated to higher titers in MDCK and mouse cell lines compared to mutants lacking the HA mutation (Figure 4F,G,K). These results suggest that while all three mutations in PB2, PA, and HA each contribute to the ability of H7N9-PBC virus to replicate better in canine, mouse and human cell lines, the HA mutation is the most important out of the three.

Since HA plays an important role in receptor binding and entry, we also investigated whether the HA mutation changed the receptor specificity of H7N9-PBC RG mP2. To examine receptor specificity, turkey red blood cells (TRBCs) were desialylated and enzymatically resialylated with either α-2,3-linked SA or α-2,6-linked SA. We compared the ability of H7N9-RG, H7N9-PBC, H7N9-PBC mP2 and the single mutant H7N9-PBC HA viruses to agglutinate native TRBCs, desialylated TRBCs mock-treated with PBS, and the two types of resialylated TRBCs (Table S1). While all of the viruses bound to both α-2,3- and α-2,6-linked SA, there was a preference by all viruses towards α-2,3-linked SA. These results suggest that the HA mutation gained in H7N9-PBC mP2 did not alter the receptor specificity of the virus.

### 3.6. H7N9-PBC Viruses Replicate in a Trypsin-Independent Manner

Polybasic HA cleavage sites are typically associated with increased virulence when found in avian influenza viruses, allowing HA activation by host proteases with widespread tissue distribution in contrast to viruses containing a monobasic HA cleavage site that is recognized by proteases found restricted to the respiratory tract. As a consequence, viral replication may spread to other tissues and organs in the infected animal. However, in the mouse model we did not detect infectious viral particles for either H7N9-RG mP2 or H7N9-PBC mP2 from organs other than the lung (Table 2). However, low titers of virus could be detected by RT-qPCR at days 3 and 6 in some animals in organs other than the lung and in the blood but in all cases no replicating virus could be isolated. This suggests that only limited and sporadic replication of virus occurred in extrapulmonary tissues and that the lungs remained the primary target of viral replication of H7N9-PBC mP2.

In spite of this finding, we were interested in determining whether H7N9-PBC viruses have the ability to utilize alternative cellular proteases to support efficient viral replication. In general, culturing of influenza viruses containing a monobasic HA cleavage site in MDCK cells requires the addition of TPCK-trypsin to the medium to facilitate HA activation for efficient replication to occur. For some viruses, the gain of a polybasic HA cleavage site allows virus replication in MDCK cells without the need for exogenous trypsin. To test the ability of H7N9-PBC viruses to grow without trypsin, MDCK cells were infected with each virus at a MOI of 0.001 in medium with or without trypsin, samples were collected at 24 h and 48 h pi, and viral titers were measured by TCID_50_ assay.

**Figure 5:**
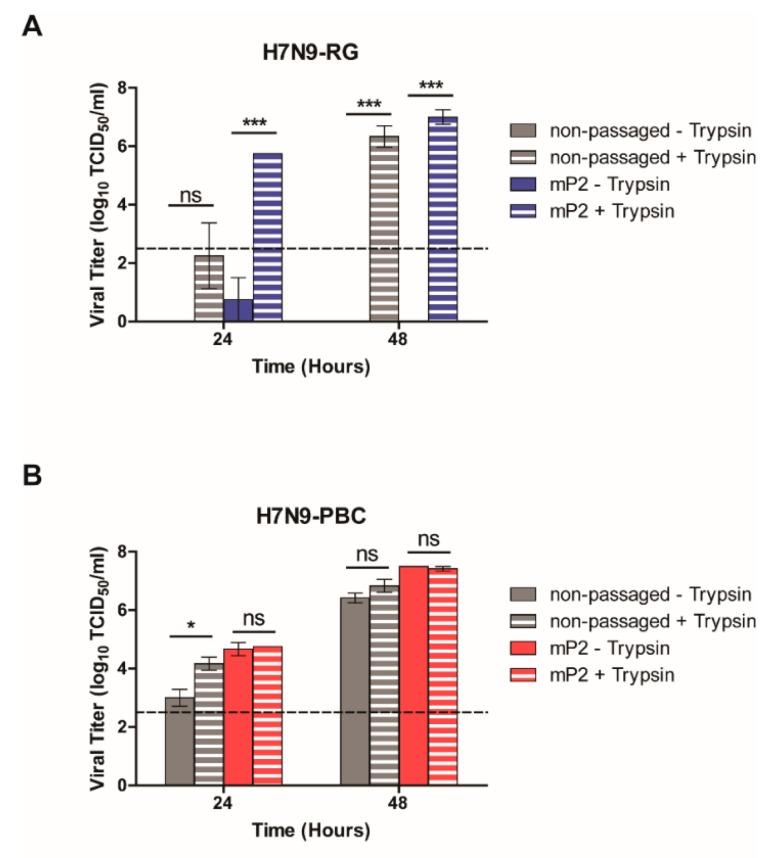
In vitro growth of H7N9 influenza viruses in the presence or absence of trypsin. MDCK cells were infected with either non-passaged or mouse-passaged (mP2) versions of (**A**) H7N9-RG or (**B**) H7N9-PBC viruses at a multiplicity of infection (MOI) of 0.001. Viruses grown in medium supplemented with 1 µg/mL trypsin are indicated. Viral titers were determined at 24 h and 48 h post-infection by TCID_50_ assay. Dotted line on each graph represents the limit of detection of the TCID_50_ assay. Mean viral titers and standard error of mean (SEM) are shown, two-way ANOVA with Bonferroni post-tests was performed, ns = not significant, *p-value < 0.05, and ***p-value < 0.001.

As expected, both the non-passaged and mouse-passaged versions of H7N9-RG virus were unable to grow without the presence of trypsin supplemented in the medium (Figure 5A). In contrast, both versions of H7N9-PBC virus replicated well even in the absence of trypsin at 48 h pi. Interestingly, at 24 h pi non-mouse passaged H7N9-PBC virus showed significantly higher viral replication when trypsin was added to the medium while the mP2 virus replicated similarly with and without trypsin (Figure 5B). This suggests that non-passaged H7N9-PBC virus may replicate slower at earlier time points when trypsin is not present and that mouse adaptation permitted more efficient recognition of the PBC by cellular proteases.

Growth without trypsin was also tested for all the H7N9-PBC viruses containing single or double mutations in either PB2, PA or HA, and like the non-passaged and mouse-passaged H7N9-PBC viruses, all the mutant viruses were able to grow efficiently without the need for exogenous trypsin in the growth medium (Figure S1). In line with the mammalian cell growth results, all H7N9-PBC mutants that contained the HA A452T mutation replicated to significantly higher levels at 24 h than the non-passaged H7N9-PBC virus, indicating a role for this mutation in replication efficiency. These results show that the PBC site in the H7N9-PBC viruses allow them to replicate well in the absence of trypsin, suggesting that these viruses are activated by alternative cellular proteases. This ability of H7N9-PBC viruses to utilize additional cellular proteases may contribute to the increase in pathogenicity observed in mice, although, the lack of viral spread beyond the lung in H7N9-PBC mP2 infected mice suggests that other host factors might be contributing to the severity of disease observed in these mice.

### 3.7. H7N9-2017PBC Virus Becomes Highly Virulent after a Single Passage in Mice

During the course of this study, a natural H7N9 isolate emerged that contained a polybasic HA cleavage site with the sequence PEVPKRKRTAR↓ (A/Guangdong/17SF003/2016). This sequence is slightly different compared to the polybasic HA cleavage site used in our study, PEIPKRRRR↓, which was derived from the PBC that naturally emerged in a large outbreak of H7N7 virus in the Netherlands in 2003 [16]. To confirm whether the naturally occurring 2017 H7N9 PBC HA sequence behaves similarly to the H7N9-PBC virus that we generated, the 2017 PBC HA site was cloned into the A/Anhui1/2013 (H7N9-RG) background virus to generate a H7N9- 2017PBC virus for pathogenesis testing in mice.

**Figure 6:**
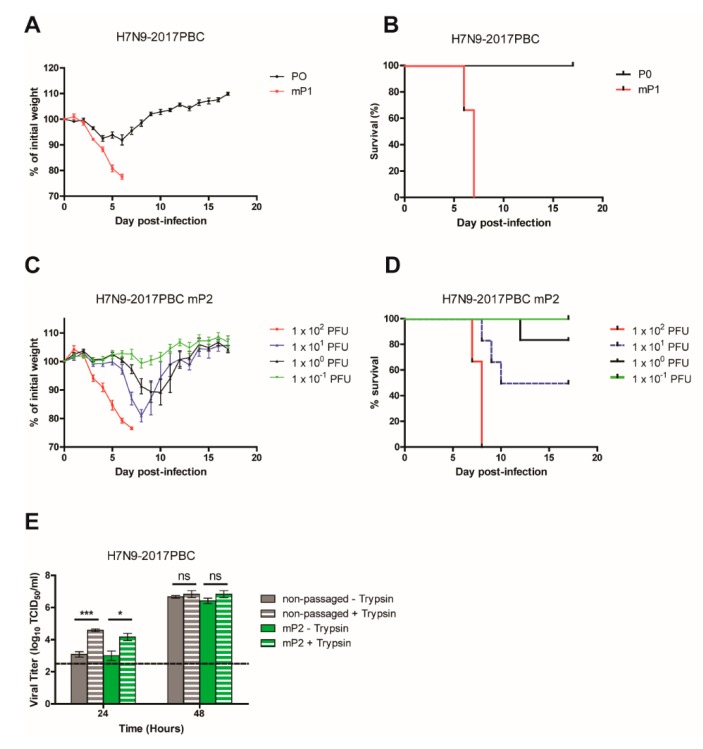
Characterization of H7N9-2017PBC virus in mice. (**A**) Weight loss and (**B**) survival of mice infected with either 3.2 × 10^5^ PFU of H7N9-2017PBC virus (P0, *n* = 6) or 50 µL of lung homogenate collected from one H7N9-2017PBC infected-mouse that was sacrificed on day 4 post-infection (mP1, *n* = 4). (**C**) Weight loss and (**D**) survival of mice (*n* = 6) infected with a dose range of 10^−1^ to 10^2^ PFU of lung homogenate from the second mouse passage (mP2) of H7N9-2017PBC virus. (**E**) In vitro growth of H7N9-2017PBC influenza virus in the presence or absence of trypsin. MDCK cells were infected with either non-passaged or mouse-passaged (mP2) versions of H7N9-2017PBC viruses at a MOI of 0.001, with 1 µg/mL of trypsin supplemented to the growth medium (+ trypsin) or without (– trypsin). Viral titers were determined at 24 h and 48 h post-infection by TCID_50_ assay. Dotted line on each graph represents the limit of detection of the TCID_50_ assay. Mean viral titers and standard error of mean (SEM) are shown, two-way ANOVA with Bonferroni post-tests was performed, ns = not significant, *p-value < 0.05, and ***p-value < 0.001.

Mice (*n* = 6) were infected IN with 3.2 × 10^5^ PFU of H7N9-2017PBC virus, the highest dose possible based on the viral stock, and weight loss and survival of the mice were monitored for 18 days pi (Figure 6A,B, P0). Initial introduction of H7N9-2017PBC virus in mice (P0) resulted in minimal weight loss and complete survival of all animals, corresponding to the non-lethal phenotype observed when H7N9-PBC virus was first introduced into mice. From the P0 group of mice, one mouse was sacrificed on day 4 pi and 50 µl of lung homogenate was prepared for passaging into four naive mice (mouse passage 1, mP1). The virulence of this mouse passaged virus (H7N9-2017PBC mP1) was assessed and like the H7N9-PBC mP1 virus, mice infected with H7N9-2017PBC mP1 experienced severe weight loss and succumbed to disease by 6–7 days pi (Figure 6A,B, mP1). Once again, a mouse was sacrificed on day 4 pi and the lung homogenate was passaged for a second time in mice (mP2). The MLD_50_ of H7N9-2017PBC mP2 virus was determined by infecting mice (*n* = 6) with a dose range of 10^−1^ to 10^2^ PFU (Figure 6C,D). A dose response to infection was observed, where animals that received the highest dose of 1 × 10^2^ PFU demonstrated severe weight loss and all succumbed to infection, while animals that received the lowest dose showed no weight loss and complete survival. From these results the MLD_50_ for H7N9-2017PBC mP2 virus was determined to be 2.2 PFU (95% CI: 1.2–3.2).

The ability of H7N9-2017PBC viruses to grow without trypsin was also examined. At 24h pi, viral replication for both non-passaged and mouse-passaged H7N9-2017PBC was significantly higher when trypsin was added to the growth medium (*p*-values of < 0.001 and < 0.05, respectively) (Figure 6E), and by 48h pi replication was similar regardless of the presence of trypsin. The H7N9-2017PBC viruses replicated slower at the earlier time point in the absence of trypsin, which contrasts H7N9-PBC mP2 viruses that replicated similarly at 24h pi without the need for trypsin (Figure 5B). By extension, the need for mouse passaging of the H7N9-2017PBC to acquire virulence does not seem to be directly related to its ability to replicate more efficiently with other cellular proteases, which include furin or other proprotein convertases that recognize the PBC site.

Viral replication of H7N9-2017PBC RG and H7N9-2017PBC mP2 in MDCK, A549, KLN 205 and DF-1 cell lines was also investigated. Similar to H7N9-PBC RG, replication of non-passaged and mouse-passaged H7N9-2017PBC was the highest in MDCK cells (Figure S2). Interestingly, while no viral titer was detected in mouse cells for H7N9-PBC RG at both time points (Figure 4B), H7N9-2017PBC RG virus was able to reach a mean viral titer of 5.4-log_10_ TCID_50_/_mL_ in mouse cells at 48h pi. Furthermore, while mouse passaging of H7N9-PBC RG virus improved overall viral replication in all three cell lines, H7N9-2017PBC replication only improved at 24h pi, with titers reaching similar levels at 48h pi with and without mouse passaging.

Despite differences in the PBC sites between H7N9-2017PBC and H7N9-PBC viruses, both viruses required a single passage in mice to bring about a highly virulent phenotype. The MLD_50_ of H7N9-2017PBC mP2 was about three-fold lower compared to H7N9-PBC mP2 (Table 1), suggesting that the naturally occurring HPAI H7N9 isolate that emerged may be slightly more pathogenic than the H7N9-PBC virus. To determine whether mouse passaging of H7N9-2017PBC virus introduced mutations similar to those found in H7N9-PBC mP2 virus, next generation sequencing of H7N9-2017PBC mP2 was completed. Interestingly, amino acid mutations were found in PB2 (Q73R, D256G) and HA (I399M) that were unique compared to those found in H7N9-PBC mP2 virus (PB2 M483K, PA D347G, and HA A452T, Table 1). Further work will be required to elucidate the importance of these mutations for virulence.

## 4. Discussion

This study demonstrated the pathogenic potential of a H7N9 avian influenza virus containing a polybasic cleavage site in a mammalian animal model. We found that host adaptation in mice required only a single passage of the virus in order to fully elicit a highly pathogenic phenotype. In addition to the PBC site in HA, we also found three additional mutations in PA (D347G), PB2 (M483K), and HA (A452T) that all contributed to increased pathogenesis in mice. Although our sequencing data was acquired using Ion Torrent NGS technology, which has a reported average error rate of 1.5% [22], by reverse genetics we confirmed the importance of each of these mutations towards H7N9 virulence. Since host adaptation occurred rapidly and the virulence associated mutations were represented in a large proportion (67%) of viruses isolated from mice, this suggests strong selective pressure for the acquisition of these mutations.

Of the three mutations, the mutation in HA (A452T) played the strongest role in increasing the virulence of H7N9-PBC virus in mice. During virus maturation, HA is proteolytically cleaved into its two mature subunits HA1 and HA2 that are important for viral fusion [23]. Consequently, mutations in HA that are known to affect virulence may affect receptor binding avidity and or preference for a particular type of receptor, such as α-2,3-linked SA for avian viruses or α-2,6-linked SA for human viruses [24,25,26]. The different receptor preference by avian and human influenza viruses is a major reason why limited transmission occurs between birds and humans, and the potential for avian influenza viruses to acquire mutations that favour receptor switching to human-virus α-2,6-SA receptors is a major concern. Recent studies have identified mutations in the receptor binding domain of H7N9 HA including G186V, K193T, Q226L and G228S, that are able to switch receptor binding from its original preference for avian-virus to human-virus receptors [27,28,29,30]. The results from our receptor specificity testing of H7N9-RG and H7N9-PBC viruses revealed that they both had a preference towards α-2,3-linked SA, with a lesser affinity towards α-2,6-linked SA. Interestingly, mouse passaged H7N9-PBC mP2 and the single mutant H7N9-PBC HA viruses retained their preference towards α-2,3-linked SA, and suggests that the A452T mutation in HA did not change receptor specificity. Amino acid residue 452 is located in the soluble ectodomain region of HA2 and mutation of alanine to threonine, which contains a slightly larger side chain, may impact the ability for viral fusion to occur. Future work to measure the efficacy of viral fusion of these viruses may help better understand the role it may play.

From the in vitro growth kinetics comparison in different mammalian cell types, we found that replication of mouse-passaged H7N9 PBC viruses replicated faster at 24h pi, with viruses containing the HA mutation replicating to higher titers in mouse and human cell lines at 24 and 48h pi. This increase in viral replication at earlier time points may explain the increase in virulence observed for the viruses that contained the HA mutation along with the PBC site. While the PB2 and PA mutations on their own each contributed to increased viral replication in mouse and human cells, the titers reached by these viruses remained lower compared to viruses containing the HA mutation. It is important to note that the H7N9-PBC RG mP2 virus had the greatest titer overall, emphasizing a requirement for all three mutations for optimal viral replication. Out of the four cell lines tested, it was not surprising that virus replication was the greatest in MDCK cells, a reason why these cells are commonly used to culture influenza viruses.

In contrast, we were surprised to detect only limited replication of H7N9 viruses in chicken cells, especially with the finding that H7N9-RG and H7N9-PBC viruses demonstrated a preference for avian-type α-2,3-linked SA. A number of reasons may explain this result including a deficiency in cell factors needed to support efficient H7N9 virus replication in this cell line, limited viral receptor availability on the cell surfaces, or poor replication at 39 °C, required for maintenance of chicken cells, compared to 37 °C for the other cell types. Some mutations in polymerase proteins can confer an adaptive advantage for avian viruses in mammalian hosts which may have included better replication at lower temperatures in a mammalian host. For example, the PB2 E627K mutation is known to increase virus replication at lower temperatures that are typically found in the upper respiratory tract of humans, and consequently can increase virulence and possibly the transmissibility of the virus as well [31]. This mutation is important for human adaptation as human clinical isolates infected with either avian influenza H5N1, H7N9, or H7N7 viruses generally contain PB2-627K, while H7N9 and H7N7 viruses collected from avian samples have PB2-627E [32,33,34,35,36,37]. Importantly, the A/Anhui/2013 (H7N9) strain used in our study already contains PB2-627K, which suggests it may already be adapted for efficient replication in mammalian cells, and may explain why these viruses replicated better in the cell lines that are cultured at 37 °C rather than the chicken cells that are grown at 39 °C. While it remains unknown what effect the PB2 M483K mutation has on polymerase function, it is clear from these results that it is contributing to increased viral replication at earlier time points in human and mouse cells, thus contributing to the overall virulence of the virus that contains this mutation.

A number of studies have also identified mutations in PA that have been shown to affect replication and or virulence [38,39,40,41]. PA encodes for an endonuclease that cleaves the cap structures off of cellular mRNA needed for viral replication [42,43]. A previous study identified a D347N mutation in the PA of H9N2 avian influenza viruses to be important for mouse adaptation [41]. In a subsequent study, both PA-A343S and PA-D347E mutations were identified in an avian H5N1 virus and shown to increase replication and virulence in mice [38]. Here we also identified the amino acid residue at 347 of PA to be important for virulence, specifically a PA-D347G mutation was found in the mouse-passaged H7N9-PBC virus. The discovery that amino acid 347 of PA is important across a number of different avian influenza viruses is interesting, and the mechanism through which this mutation influences virulence will need to be studied further.

The recent isolation of HPAI H7N9 influenza viruses containing a PBC site is a major concern [11,44]. Our study demonstrates that H7N9 influenza viruses bearing a PBC from either a naturally occurring highly virulent H7N7 virus or the naturally occurring H7N9-2017PBC are both highly pathogenic in mice after a single passage. Subtle differences did exist between these two PBC containing H7N9 viruses, such as a lower MLD_50_ value for H7N9-2017PBC and slower replication at 24h pi in the absence of trypsin. Interestingly, sequencing of the mouse-passaged H7N9-2017PBC virus revealed a different set of mutations in addition to the PBC in HA, such as PB2-Q73R/D256G and HA-I399M. The two mutations PB2-Q73R and HA-I399M have not been described previously; however, the D256G mutation has been described to enhance viral polymerase activity in vitro and was important for replication of an avian H5N1 virus in pigs [45]. It was surprising that different mutations arose from mouse passaging of this closely related H7N9-2017PBC virus. However, it is possible that the slight difference in viral replication in the absence of trypsin at early time points suggests that recognition of this PBC site by alternative proteases may differ compared to recognition of the PBC site in H7N9-PBC virus. The efficiency of replication early on during infection in an animal model may impact the mechanism through which the virus adapts itself to the host to gain increased virulence, and may explain why different mutations appeared after mouse passaging of each virus. Further work will be required to determine the importance of these mutations for H7N9-2017PBC virulence.

Other studies have examined the virulence of natural isolates of HPAI H7N9 from humans or chickens in animal models including mice, ferrets, and non-human primates [44,46,47,48]. Interestingly, Imai et al. showed that human isolated HPAI H7N9 is lethal in mice, partially lethal in ferrets, and non-lethal in cynomolgus macaques without prior adaptation in these animal models [46]. In contrast, our H7N9-PBC and H7N9-2017PBC viruses required a single passage to become lethal in mice. This discrepancy could be attributed to our use of a 2013 human isolate (Anhui) of LPAI H7N9 to generate our H7N9-PBC viruses compared to the 2016 HPAI H7N9 human isolate used by Imai et al. [46]. The extra years of circulating in avian populations by the 2016 H7N9 human isolate has likely driven the acquisition of mutations, in addition to the PBC site in HA, which confer to it an adaptive advantage in mammals which permits efficient replication in mice and causes rapid lethal disease.

Similarly, a 2017 human isolated HPAI H7N9 was shown to cause immediate lethal infection in mice, however, a HPAI H7N9 virus isolated from chicken was not lethal in mice [47]. Shi et al. showed that mice infected with a 2017 chicken-isolated HPAI H7N9 resulted in a non-lethal infection, although virus replication was detected in the nasal turbinates and lungs [49]. They further showed that inoculation of this same virus into ferrets was also non-lethal, however viruses sequenced from the organs and nasal washes acquired a PB2-627K or a PB2-701N mutation [49], both known to be critical for mammalian adaptation [32,33,50,51]. Shi et al. then used the ferret-passaged virus (containing either 627K or 701N) to infect mice and found that both viruses resulted in severe and lethal disease [49]. Another study showed different strains, including HPAI and LPAI H7N9 viruses, presented diverse biological characteristics and virulence for mammals [44]. This may be caused by the different adaptation progress of H7N9 for mammals. The outcome of these studies is consistent with our results, where host adaptation of HPAI H7N9 is required to cause severe disease and that this adaptation occurs quite rapidly. Interestingly, our HPAI H7N9 virus already contained the PB2-627K mutation, yet it still required additional mutations in HA, PB2, and PA for complete virulence, suggesting that a combination of factors is required for mammalian adaptation of these viruses.

Avian H7N9 influenza viruses are a major concern to public health, and the emergence of a naturally occurring HPAI H7N9 a few years after the first human case highlights the potential of these viruses to quickly gain virulence factors. Although previous studies have shown that the presence of a PBC in HA is not always sufficient for high virulence [52], here we confirm that the polybasic HA cleavage site for H7N9 viruses contributes to increased pathogenicity in mice. Host adaptation was necessary for increased virulence and occurred rapidly by these viruses, causing severe disease in mice after a single passage. Here we have also identified a novel HA mutation (A452T) that plays a critical role in virulence of HPAI H7N9 viruses in mice. Further studies to characterize the mechanisms of the mutations we identified towards virulence and assessment of the pathogenic potential of these HPAI H7N9 viruses in other animal models, such as chickens or ferrets, will help provide a better understanding of what these viruses need to replicate well and cause disease in hosts. Careful monitoring of these viruses as they evolve in current H5/H7 vaccinated poultry populations and the continued development of new countermeasures against these viruses will be important for better pandemic preparedness.

## Figures and Tables

**Table 1:** Determinants of pathogenicity of mouse passaged H7N9-PBC. Next-generation sequencing results of H7N9-PBC viruses after two passages in mice identify amino acid mutations in PB2 (M483), PA (D347), and HA (A452, H7 numbering with signal peptide included) genes. Amino acid residues mutated are noted and the percent frequency of the mutation observed is shown. A complete reverse genetics engineered H7N9-PBC mP2 containing all three identified mutations was created (H7N9-PBC mP2-PB2/PA/HA) along with H7N9-PBC viruses containing all possible combinations of the mutations found. The mouse median lethal dose (MLD_50_) was determined for all viruses by infecting mice (*n* = 6) starting at 1 × 10^4^ PFU and subsequent 10-fold serial dilutions. For * and ^#^, MLD_50_ was determined previously starting with maximum dose possible based on viral stock titer. ^+^ MLD_50_ was determined using the method of Miller and Tainter, 95% confidence interval (CI) values are included.

Virus	Origin	PB2	PA	HA	HA Cleavage Site	MLD50 (PFU)	95% CI^+^
A/Anhui/1/2013 (H7N9-RG)	Reverse genetics	M483	D347	A450	PEIPKGR↓GL	2.04 × 10^4^*	
A/Anhui/1/2013 (H7N9-PBC)	Reverse genetics	M483	D347	A452	PEIPKRRRR↓GL	> 3.7 × 10^6#^	
H7N9-PBC mP2	Mouse-passaged lung	M483K (65%)	D347G (68%)	A452T (66%)	PEIPKRRRR↓GL	7.0 × 10^0^	6.0–8.0 × 10^0^
H7N9-PBC mP2-PB2/PA/HA	Reverse genetics	M483K	D347G	A452T	PEIPKRRRR↓GL	1.0 × 10^1^	9.0–1.1 × 10^1^
H7N9-PBC PB2		M483K	D347	A452		> 1.0 × 10^4^	
H7N9-PBC PA		M483	D347G	A452		> 1.0 × 10^4^	
H7N9-PBC HA		M483	D347	A452T		2.78 × 10^2^	2.7–2.9 × 10^2^
H7N9-PBC PB2/PA		M483K	D347G	A452		> 1.0 × 10^4^	
H7N9-PBC PB2/HA		M483K	D347	A452T		2.22 × 10^2^	2.20–2.23 × 10^2^
H7N9-PBC PA/HA		M483	D347G	A452T		1.02 × 10^2^	1.01–1.03 × 10^2^

**Table 2:** Replication of H7N9-RG and H7N9-PBC mouse-passaged viruses in mice. Groups of mice (*n* = 5) were infected intranasally with 10^2^ PFU of either H7N9-RG mP2 or H7N9-PBC mP2 viruses, blood and tissues were collected on days 3, 6, and 8 post-infection. Viral load was first measured by RT-qPCR detecting the M gene, with mean Ct values reported, and the limit of detection of the assay was 50 genome copies/mL or /g tissue. For all RT-qPCR positive samples, infectious viral loads were determined by TCID_50_ assay, where x is the number of samples that were RT-qPCR positive, and the mean logTCID_50_/_mL_ or /g ± standard error of mean is shown. ND = not determined.

Virus	Sample	Day 3		Day 6		Day 8	
		Mean Ct (x / 5 mice)	LogTCID_50_ per ml or g (*n* = x)	Mean Ct (x / 5 mice)	LogTCID_50_ per ml or g (*n* = x)	Mean Ct (x / 5 mice)	LogTCID_50_ per ml or g (*n* = x)
**H7N9-RG mP2**	Blood	0	ND	0	ND	0	ND
	Heart	33.5 (2)	(0/2)	34.6 (1)	(0/1)	0	ND
	Spleen	0	ND	0	ND	0	ND
	Kidney	0	ND	0	ND	0	ND
	Intestine	0	ND	0	ND	0	ND
	Lung	24.3 (5)	3.3 ± 2.7 (3/5)	18.6 (5)	6.0 ± 0.5 (5/5)	27.9 (5)	1.8 ± 2.3 (2/5)
	Eyes	0	ND	0	ND	0	ND
**H7N9-PBC mP2**	Blood	32.5 (2)	(0/2)	0	ND	27.5 (2)	(0/2)
	Heart	30.9 (3)	(0/3)	31.8 (4)	(0/4)	33.9 (3)	(0/3)
	Spleen	34.3 (3)	(0/3)	34.4 (1)	(0/1)	0	ND
	Kidney	30.7 (1)	(0/1)	34.7 (1)	(0/1)	0	ND
	Intestine	34.9 (1)	(0/1)	35.6 (1)	(0/1)	0	ND
	Lung	15.7 (5)	7.3 ± 0.4 (5/5)	15.6 (5)	7.1 ± 0.3 (5/5)	23.3 (5)	6.2 ± 0.2 (5/5)
	Eyes	0	ND	34.0 (2)	(0/2)	0	ND

## Supplementary Materials

The following are available online at https://www.mdpi.com/1999-4915/12/1/65/s1, Figure S1: Trypsin dependent growth of H7N9-PBC viruses containing mutations in either PB2, PA, or HA., Figure S2: Growth of non-passaged H7N9 2017-PBC RG and mouse-passaged H7N9 2017-PBC mP2 influenza viruses in mammalian cell lines. Table S1: H7N9 influenza virus hemagglutination titers in turkey red blood cells.
