# Supplementary material for: H7N9 Influenza Virus Containing a Polybasic HA Cleavage Site Requires Minimal Host Adaptation to Obtain a Highly Pathogenic Disease Phenotype in Mice
Source: Viruses. 2020 Jan 5;12(1):65. doi: 10.3390/v12010065 (PMC7020020; doi:10.3390/v12010065)
Supplement: Supplementary file 1 [file viruses-12-00065-s001.zip › viruses-605935-suppl/Figure S1.pdf]

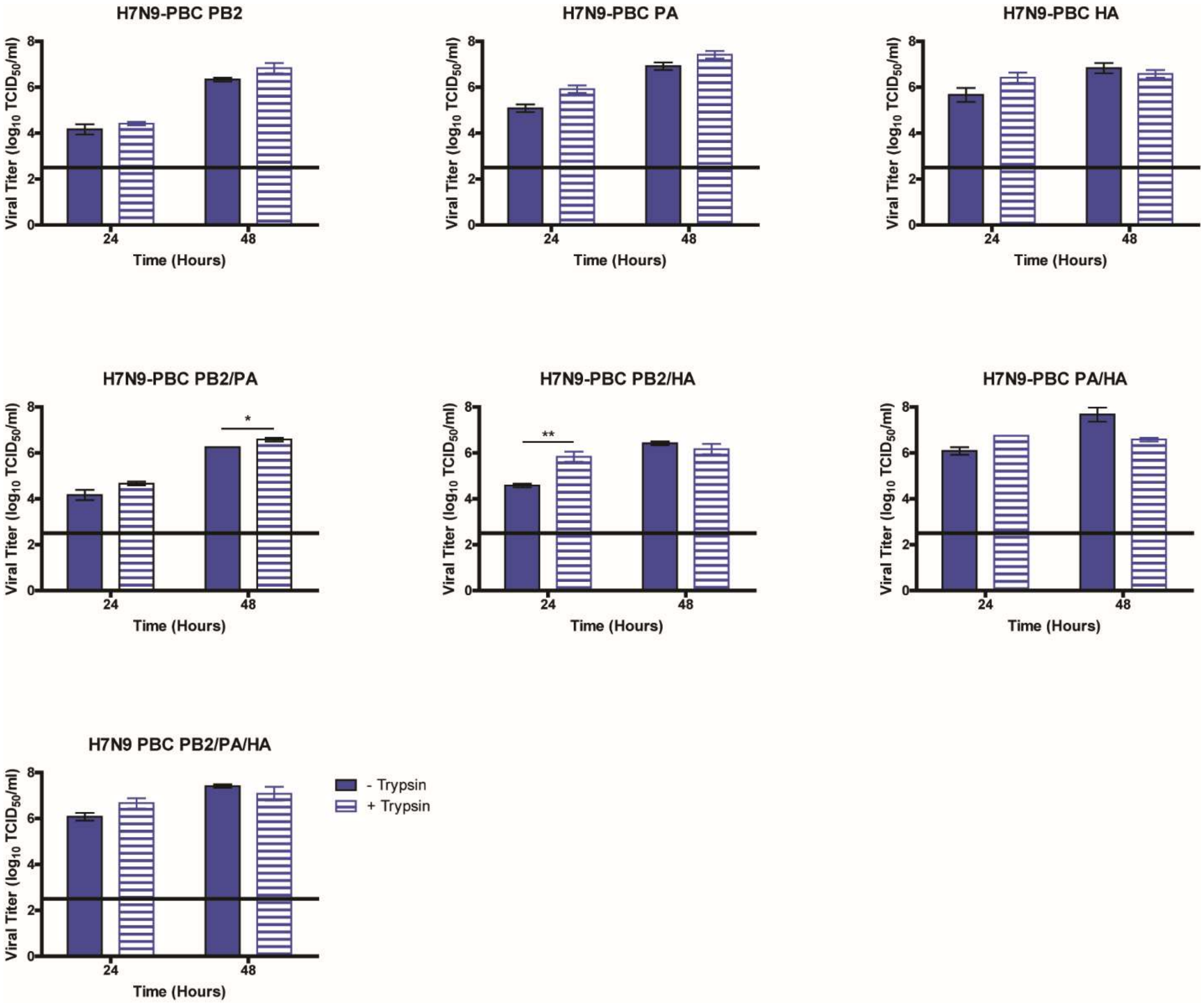

Figure S1. Trypsin dependent growth of H7N9-PBC viruses containing mutations in either PB2, PA, or HA. MDCK cells were infected with each virus at a MOI of 0.001, with 1 µg/ml of trypsin supplemented to the growth medium (+ Trypsin) or without (- Trypsin). Viral titers were determined at 24h and 48h post-infection by TCID50 assay. Dotted line on each graph represents the limit of detection of the TCID50 assay. Mean viral titers and standard error of mean (SEM) are shown, 2-Way ANOVA with Bonferroni post-tests was performed, *p-value <0.05, and **p-value < 0.01.
