# Supplementary material for: H7N9 Influenza Virus Containing a Polybasic HA Cleavage Site Requires Minimal Host Adaptation to Obtain a Highly Pathogenic Disease Phenotype in Mice
Source: Viruses. 2020 Jan 5;12(1):65. doi: 10.3390/v12010065 (PMC7020020; doi:10.3390/v12010065)
Supplement: Supplementary file 1 [file viruses-12-00065-s001.zip › viruses-605935-suppl/Figure S2..pdf]

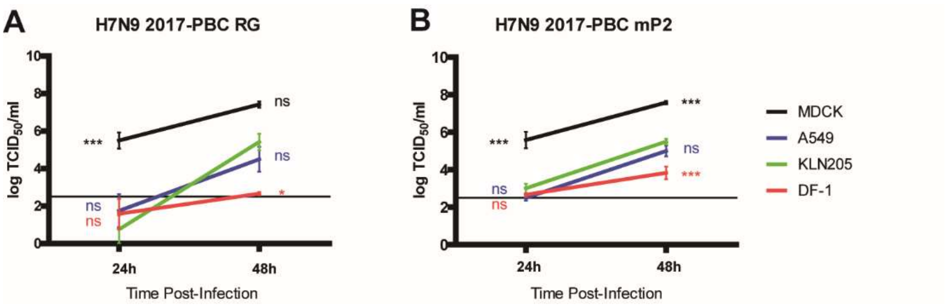

Figure S2. Growth of (**A**) non-passaged H7N9 2017-PBC RG and (**B**) mouse-passaged H7N9 2017-PBC mP2 influenza viruses in mammalian cell lines. Viral replication was determined in canine kidney (MDCK), human lung (A549), mouse lung (KLN 205), and chicken fibroblast (DF-1) cell lines at 24 and 48 hours post-infection with 0.5 µg/ml TPCK-trypsin supplemented into the culture medium. Viral titers were determined by $TCID_{50}$ assay using MDCK cells with 1.0 µg/ml TPCK-trypsin in the medium. Dotted line on each graph represents the limit of detection of the $TCID_{50}$ assay. Mean viral titers and standard error of mean are shown, 2-Way ANOVA with Bonferroni post-tests was performed comparing all results to KLN 205 at each time point, ns = not significant, * or ** or *** denotes p-values of < 0.05, or < 0.01, < 0.001, respectively.
