# Supplementary material for: H7N9 Influenza Virus Containing a Polybasic HA Cleavage Site Requires Minimal Host Adaptation to Obtain a Highly Pathogenic Disease Phenotype in Mice
Source: Viruses. 2020 Jan 5;12(1):65. doi: 10.3390/v12010065 (PMC7020020; doi:10.3390/v12010065)
Supplement: Supplementary file 1 [file viruses-12-00065-s001.zip › viruses-605935-suppl/Table S1.pdf]

Turkey Red Blood Cells	Virus HA Titer			
	H7N9-RG	H7N9-PBC	H7N9-PBC mP2	H7N9-PBC HA
**Native**	16	16	64	32
**Desialylated and PBS treated**	0	0	2	1*
**α-2,3-resialylated**	64	8	128	64
**α-2,6-resialylated**	4	2	64	8

Table S1. H7N9 influenza virus hemagglutination titers in turkey red blood cells. Turkey red blood cells were either left untreated (Native), desialylated with receptor destroying enzyme and treated with PBS or resialylated with either α-2,3-linked Neu5Ac or α-2,6-linked Neu5Ac. For each virus, 2-fold serial dilutions were prepared with PBS, and virus dilutions were mixed with an equal amount of untreated or treated 1.5% suspension of turkey red blood cells. Hemagglutination (HA) titers were read after 30 min at room temperature and are reported as the reciprocal of the lowest dilution showing complete agglutination. A value of 1* represents the neat viral stock.
